# Stopping implementation of administrative decisions in Saudi regulation

**DOI:** 10.1016/j.heliyon.2021.e07638

**Published:** 2021-07-21

**Authors:** Noor Issa Alhendi

**Affiliations:** Department of Public Law, Faculty of Law, Applied Science Private University, Jordan

**Keywords:** Administrative decision, Board of grievances, Urgency condition, Seriousness condition

## Abstract

This study deals with stopping implementation of administrative decisions in Saudi regulation, as it aims to clarify the concept of stopping implementation of administrative decisions, and their justifications, as well as clarifying the conditions for it that are contested on the grounds of cancellation. Execution of the contested administrative decisions on the grounds of cancellation, through a statement of the timeliness of the judgment issued in the request to stay the implementation of the decision, its evidence, and its appeal.

To clarify this issue, the study will depend on the descriptive and analytical method to describe and analyses legal texts, judicial decisions and jurisprudential opinions related to the subject of the study.

The study concluded that the request to stop implementation of the contested decision on the grounds of cancellation is an urgent request submitted to the Saudi Board of Grievances to protect the interests of individuals and employees from management decisions, the privileges and powers granted to it from legal texts to achieve the public interest, until the final decision is made in the annulment case. An exception to the general rule, because the implementation of administrative decisions has consequences that harm some individuals and employees to whom the decisions of the public administration address, so they are entitled to an exception to submit a request to temporarily suspend their implementation.

The department competent to decide on a request to stop implementation of administrative decisions does not respond to this request except when certain objective conditions are met, which the administrative judiciary is strict in their availability, which is the condition of urgency, and the annulment lawsuit is based on serious reasons. Therefore, with the availability of these conditions, the Saudi administrative judiciary has the right to stop implementation of administrative decisions through the judicial decision issued in this request.

In its judicial decision, the Board of Grievances emphasizes the temporary nature of the judicial decision issued in the request for a stay of execution, as is the case for summary judgments, in addition to that it is a final judgment that has the elements and characteristics of judgments, so it possesses the power of the thing judged in the order in which it was issued. Temporary judicial proceedings pending a decision on the annulment lawsuit, as it possesses relative authority due to its urgent nature.

Finally, if the court ruling in the annulment case is rejected; the judgment issued for the stay of execution expires and becomes as if it was not issued, but if the judicial judgment is issued to cancel the administrative decision, this means the continued enforcement of the judgment of stay of execution.

## 1 Introduction

The importance of this study appears in that it deals with the Saudi legal texts which related to suspend implementation of the contested administrative decisions in the case of judicial annulment in the Board of Grievances in Saudi Arabi kingdom.

The request to stop implementation of administrative decisions is considered a guarantee to stop the public administration from tyranny, arbitrariness and to go to implement its decision during the appeal procedure, which leads to serious harm to individuals and employees to whom the administrative decision addresses.

So, the study questions are: What is the meaning of the request to suspend implementation of the contested administrative decisions by cancellation by Board of Grievances?

What are the conditions for suspending implementation of administrative decisions contested by cancellation?

What is the judicial ruling issued in the request to suspend the implementation of the contested administrative decisions to cancel?

To clarify this issue, the study will rely on the descriptive-analytical method to describe and analyses the statutory texts, judicial decisions and jurisprudential opinions related to the subject of the study.

The problem of this study is that the legal texts did not adequately address the suspension of the implementation of administrative decisions by Board of Grievances. There is a question about whether these texts balance the rights of individuals from direct implementation of administrative decisions and the public interest when implementing these decisions. In other words, have the Saudi legal texts established a balance between the interest of the public administration in continuing to implement its administrative decisions, and the interests of individuals and employees by protecting them from the effects that are difficult to compensate for if those decisions are implemented? Especially if the administration implements its decision immediately - subject to appeal - so that the appellant has no chance of getting any benefit from stopping these decisions.

## 2 The concept of stopping implementation of administrative decisions

The administration practices authority by issuing administrative decisions, that immediately after issuance become valid and entertain a direct executive power, therefore it can oblige the individual to conform with its decision without a permission from the administrative jurisdiction. ‘This is considered as characteristic of the administrative authority’ (Shatanawi, 2002) where the administration by issuing the decision puts the legal text into implementation. This implies being convinced that the decision should be understood as correct and legitimate, this is the general concept (Yousofy, 2017).

Though these decisions may occasionally be stained with a flaw that renders them illegitimate. This illegitimacy will push the party of concern to file an appeal before the Board of Grievances through a 'case of cancellation' that requests canceling the administrative decision and this case may be accompanied with an urgent request to suspend these decisions until being investigated. Basically, the case of cancelation does not prevent the administration from going forward in the execution of the decision and arrange all its consequences by using the prerogative of direct execution that they entertain, while going against that will lead to the paralysis of the activity of the administration that is concerned with the public interests. Therefore, as an exception (Khalifah, 2008), the Saudi legislator in article (9) of the ‘Code of Litigation Before the Board of Grievances’ permitted the administrative court to suspend the decision under appeal if it is estimated that the execution will result into irreversible consequences had the court cancelled the decision of the appealed case (Baloushah, 2016). Therefore, it was stated that "raising a case of cancelation does not consequently lead to the suspension of the decision, though it is permissible that the court can give an order of suspension – if being requested to do so, seeing that the execution will result into irreversible consequences – until a ruling takes place. Article (5/8) of the same code stated that "Exceptional to what is mentioned in the previous paragraph, the court may accept a case of cancelation in the duration of informal grievance (grievance outside the court like discussing the case with the employer etc.) in urgent cases when accompanied with a request to suspend the administrative decision being under request of cancelation, provided that there is grievance directed to the body that issued the decision, then the court will immediately issue a request to suspend the execution, and the case will be looked into at the end of the duration given to informal grievance, or if the body that issued the decision refused the grievance before end of duration.

Consequently, the request for suspending the administrative decision under appeal is defined as "not commencing the implementation of the decision within a duration, because of the occurrence of one of the causes of suspension, usually the rise of a conflict aimed to gain a verdict that cancels the execution" therefore, a request to suspend the decision is preventive against risks in continuing surrendering to an execution subject to cancelation by the court, and consequently against the risk of irreversibility, and other undesirable results that will come out as a result of executing the decision^.^

It is inevitable to indicate that filing a request to suspend a decision is not carried out spontaneously by the judge, therefore it must be filed by the opponent as being a request related to the original request and stemming out of it, to temporarily protect the interest of the claimant until a verdict is reached regarding the case. Article (5/8), code of litigation before the Board of Grievances asserted in other words "When accompanied by a request to suspend the administrative decision under request for cancelation" and therefore it is not possible to file a request to suspend the execution independent of the case of cancelation.

Article (8) code of litigation before the Board of Grievances stated that "Exceptional to the regulations mentioned in the previous paragraph, the court can accept a case of cancelation in the duration of grievance in urgent cases when accompanied with a request to suspend the decision under request of cancelation provided that there is a grievance directed to the body that issued the decision.

## 3 Conditions of stopping implementation of administrative decisions

In the beginning and before examining the conditions needed to suspend the administrative decision under appeal, there must be an existing administrative decision in first place, and in the absence of the latter, the request for suspension will be baseless i.e. based on no decision, which means that the request will be revoked, and this was asserted by the Board of Grievances by giving the statement that "It is conditional to suspend the execution of the decision, where an existing administrative decision enables investigating the conditions needed to suspend the execution. Therefore, because there is no decision issued by the administration to confiscate the guarantee, then a request to suspend the execution of a decision that is not issued is baseless ... though this will have a negative effect on the claim that they were informed in an address letter implying a threat of deducting the value of debts – mentioned in the letter – from the guarantee if the sum indicated is not paid within fifteen days. This is only an urge to pay the sum as mentioned by the defense while there was no order of deduction, therefore the request is baseless because there is no decision issued, and this is the reason why the request is denied"^1^What is worth mentioning is that the Board of Grievances ordered the suspension of the decision in the same case when the administrative body sent a letter to the bank requesting the confiscation of the guarantee from the claiming company. This means that the decision is existent, and the conditions of seriousness and urgency may be existent to suspend the decision.^2^

The Board of Grievances stressed that the court should not suspend the decision unless seen from the papers, and without effecting the request of cancelation when looking at it that the suspension is based on two main elements, the first is urgency, where the execution will result in irreversible consequences, the second is related to the principle of legitimacy i.e. the case is based seemingly on serious causes – Proving that the contractor was defective in carrying out this contractual commitment allows the administrative body to withdraw the work from him and complete it on his expense if the necessities were present, then a request to suspend the decision of withdrawal and other consequences like giving the project to another contractor will not be based on right foundation, therefore the element of seriousness will be present – and initially for a request of suspension to be accepted, the decision was not actually executed, otherwise the request for suspension will be baseless^.^

### 3.1 Urgency condition

The board of grievances explained the concept of 'urgency' that leads to suspending the decision under appeal, where in one of the verdicts stated that "and because the defendant buried the waste in this place, consequently will cause harm to the surrounding environment, stretching its harm to the health of humans, animals and plantation, and it may be not possible to mend any harm that results from burial because the soil is impregnated with radiations"^3^ and it ruled in another verdict that "because executing the decision under appeal will result into irreversible consequences, a procedure will end up with stopping the execution of the decision of removal and return the electric power according to article (9), code of litigation before the Board of Grievances, therefore the department orders stopping the execution of the decision issued by the principality of Riyadh no. …….. the removal of structures that belong to the claimant in this case (Thnaibat, 2013), If it is conditional to stop the administrative decision, that the execution may result into irreversible effects and most of the effect is materialistic, then it can also be ruled to stop the decision if those effects were significant, either alone or with other effects, and this was stressed by the Board of Grievances where it stated that "In addition to the enormous harm that may result from the implementation of the decision of the committee, the most critical may be the exacerbation of the conflict between the two neighbors before the ruling, and the psychological suffering that befalls on the claimant, which may propel him to actions of no desirable consequences.^4^The conflict was about a request to stop the decision of the committee that requires removing the trespasses represented by a swimming pool, a shade, and a carpark in the house of the claimant; a response to a claim raised by his neighbor. This deserves urgent action if carrying out the decision under appeal will result in irreversible effect, even though a verdict of cancelation follows, the expression 'irreversible consequences' came generic, therefore it can be either materialistic or significant, Then it was explained that "carrying out the removal, then comes a ruling that stops the removal will lead to the unrest of the family of the claimant, and alternatively it will destabilize his employment status and will have an impact on his work capabilities and his performance" The previously mentioned expressions clearly refer to significant harm.

It is important to know that the verdicts of the Board of Grievances expanded – in stopping administrative decisions – to domains where the administration entertains a wide discretional authority including transfer of employees. The Board of Grievances issued an order that stopped a decision that ordered the transfer of one of the employees after being sure of the presence of conditions required to stop the decision and stated that "…. In addition to what may result from working at … of enormous harm that cannot be mended, where the most serious harm will be because of his extreme short sight as indicated in the medical report that would exacerbate his psychological suffering which will result from being away from his family where he is in dire need for them, especially at this time because of his incapability in managing himself without help from his family due to his short sight, and as the claimant said, this may drive him to resignation. This is what ensures that the decision may be stopped even though the irreversible harm that may result from the decision is neither materialistic nor financial, and it is enough to be significant or physical as mentioned in the previous verdict.

### 3.2 Seriousness condition

What is meant by serious reasons is the illegitimate faces of the administrative decisions, i.e., the reasons that the request for suspension are based upon, and these reasons are strong enough to guarantee cancelling the decision. The judge is the one who would estimate this seriousness, therefore looking into documents and evidence that support the request to create an initial idea about the degree of legitimacy of the decision, then giving an order to stop the execution as a temporary procedure that will not affect the base of the right (Aljailani, 2017). And it was stated in a verdict by the Board of Grievances that "apparently from the papers stems out – from the decision under appeal – a violation of the law, where it is probable that when it is present, there will be a verdict of cancelation after looking into the case".^5^

It is inevitable to indicate that the Saudi legislator – in article (9) code of litigation before the Board of Grievances – did not state the condition of seriousness, though this condition is tangible in most verdicts, where it stipulates to accept the request for suspending the decision two conditions: presence of urgency, and the request of the claimant be apparently – from the papers – based on true causes.^6^And what insures that, the Board of Grievances ruled in this regard that "the urgent request – by the claimant – to suspend the execution of the negative decision under appeal where the city council abstained from renewing the bakery license is rejected because of the absence of the element of seriousness"^7^ In another verdict it referred to the conditions necessary to apply the state of suspension "The court must not suspend the an administrative decision unless apparently seen from the papers and without effecting the base of cancelation request at the time of ruling, that the request for suspending is based on … related to the principle of legitimacy, that the claim of the requester based on serious causes. And the Board of Grievances doesn't consider the public interest as a condition for issuing a judicial verdict, and its verdicts settle on the necessity of the presence of two conditions, the condition of urgency and the condition of seriousness, but the public interest may affect the judge's decision, and in one of its verdicts it was stated that "An imminent danger is over there, the parties related to the case when working together …and the tribal conflict that may happen, and if one of the parties kept working in the land, it may be harmful to him, or to the trespasser or to the treasury and such harm may be irreversible.^8^

## 4 The judicial verdict issued regarding the request for stopping an administrative decision under appeal

The administrative body is committed to comply with the judicial verdict ordering the suspension of the decision under appeal, and they should stop continuing the execution. If the administration continues carrying out the execution despite the issuance of an order to stop, then this is considered as coercion and they will be forced to pay compensation, in addition to being a criminal offence described as refraining from executing a judicial verdict (Thnaibat, 2013). Therefore, this research will deal with the 'time scheduling' (defining timelines) of the judicial verdict issued to suspend the execution of the decision, its 'degree of power' and 'applicability' in the next requests as follows:

### 4.1 Time scheduling of the judicial verdict issued to suspend a decision under appeal

A judicial verdict that orders the suspension of a decision under appeal until ruling, is considered a temporary verdict, but this character doesn't bar it being considered as conclusive in what it has ruled, and this is related to the degree of power of the decision,^9^ and it doesn't restrict the court when looking into the case of cancelation, i.e. the issuance of a verdict that suspends the decision doesn't mean that the decision will be cancelled, and rejecting a request for suspending it doesn't mean that the court will reject the case of cancelation (Shatanawi, 2004), and if the court ruled that the case is rejected, then the decision of suspension will be void, and the administrative decision will be executed with all of its effects (Baloushah, 2016). Therefore the verdict regarding the case subject will void any legal value regarding the verdict issued to suspend the decision under appeal, and the reason for considering stopping the execution as temporary, is the issuance according to the examining of the apparent look of papers and documents to confront a condition where the decision is under appeal, and the task of examining the legitimacy is left to the court when they look into the case of cancelation (Khalifah, 2008). The court deeply examines the evidence and the documents to decide whether the administrative decision is appropriate or not (Salih, 2011).

It is of no doubt that the issuance of a verdict in the cancelation case has a direct impact on the decision issued previously in the request of suspending the decision either the verdict was to rescind the decision or reject the case from the beginning (Abdulwahid, 1984). If the verdict is issued to cancel the decision, then it uproots it from the date of issuance, i.e. the verdict of suspension will continue being in effect because the latter is only a temporary stopping of the decision until a verdict is reached in the case of cancelation, therefore a verdict that cancels the decision means assurance of what previously was issued in suspending the decision, that the decision exhibits a flaw that led to being void, i.e. the verdict of suspension was correct according to the causes of seriousness that led to canceling the decision under appeal (Beliani, 2001). In addition, the administration is obliged to return the status to what it was before the issuance of its flawed decision, where its suspension may only be targeting the suspension of execution without a request to return the status to what it was before, therefore the issuance of a cancelation verdict is considered a termination of the verdict of suspension and uprooting its legal existence, then the beginning of action according to the cancelation verdict (Abdulwahid, 1984), while if a verdict is issued rejecting the cancelation case, then the effect of the issued verdict suspending the execution will terminate, where the executive power of the decision will be retained despite the absence of a text that states so. ‘The Board of Grievances ruled in one of its rulings that “there should be a state of urgency that calls for deciding the sub-request due to the presence of irreversible consequences if the decision is postponed until a decision is made on the original subject matter of the case’^10^.

Finally, here are some judicial implementations by the Board of grievances (Alsuailim, 2012) emphasizing that the decision issued by the administrative judge in a request to suspend the administrative decision under appeal is a judicial verdict in an existing conflict represented in an urgent sub-case strongly related to the case of cancelation, therefore the request of suspension is an urgent one that requires a ruling in the case (Khalifah 2008), consequently the verdict issued in this request is temporary.

Judicial verdict no. (30/decision/a/22 year 1427h) in the request of the case of cancelation no. (274/5/decision year 1427h) it was a request of suspension regarding the decision issued by the defendant which bars the claimant from sitting for the exams in the second semester, therefore the judicial department suspended the decision and allowed the defendant to sit for the exams, because not sitting for the exams and the delay in getting her results will result in missing the deadline for university registration and this will not be reversible.

- Judicial verdict no. (64/decision/a/5-year 1427h) In the urgent request in case no. 2804/1/decision year 1427h. This request was raised by the claimant, a company contracting with the administrative section concerned with marketing and promotion of commercial advertising exclusive of the Saudi Television for three years, where the administrative contracting party asked the claimant to pay the rest of the first and second installments, and the minimum value of the third and fourth installments …. With the threat that if they do not comply then they will confiscate the bank guarantee, withdraw the contract, and do the work on their expense, therefore the claimant requested the suspension of confiscation. The department submitted as a condition of urgency, that the dues of the ministry are less than the sum requested by the claimant and confiscating the guarantee will cause harm to the position of the company and to its commercial reputation, had this happened the harm will be irreversible. It was added that the Board of Grievances settled for subjecting the procedures of suspension – in matters related to the contract – to the text of article (7) code of litigation and procedures, and they ended up with the suspension of actions that would lead to the confiscation of the guarantee.

### 4.2 The degree of power of a judicial verdict issued, regarding suspending a decision under appeal

The judicial verdict issued requesting suspension entertains a power of special nature, not limited to the subject of the decision, and not limited to what is subject to ruling without restricting the ruling in the case of cancelation. Article (32) code of litigation before the Board of Grievances stated "verdicts issued to cancel, have power over the public" therefore, a verdict issued suspending a decision under appeal gains absolute power over the public, while a verdict issued rejecting this request gains partial powers limited to the parties of the case. Likewise if a verdict issued rejecting a cancelation has a partial power, then the power of a verdict rejecting a request of suspension will not exceed the domain of this partiality (Abdelbaset, 2007) - this goes back to the tie between the request for suspension and the case of cancelation – where the domain of power is limited to the subject of the ruling and whatever initial issues ruled in, while the court doesn't restrict itself to this power when looking into the case of cancelation (Khalifah, 2008).

- Power of the verdict suspending a decision regarding its subject.

A request to stop the execution of the decision under appeal is represented by the suspension of execution until a ruling is reached in the case of cancelation. The degree of power that this verdict has, assumes that the court will adhere to what was issued in this regard, therefore it is not permissible for the court to back off, and it is not permissible for the party of concern to raise the conflict again before the court if the circumstances that led to the issuance of the verdict have not changed.

- Power of the verdict suspending a decision regarding initial issues that have been investigated.

Initial issues are represented in what is related to a request of suspending a decision in what has been submitted to the court, of defenses while examining the request and being looked at and examined by the judge, such as rejection based on non-specialty of the court, or rejection because of missing the deadline, or rejected because of non-termination of the decision under appeal. If the court judged the subject in the sub-defenses and the judgment in the request of suspending the execution acquired the power of the subject being judged and became conclusive, it will be forbidden for the court to reexamine what was already been judged. Even though the verdicts in the initial issues related to the request of suspending execution and acquired the power of the subject judged forbids the court from reexamining them, this power does not handcuff the administrative supreme court which has the right of looking into them while looking into the subjective part (Khalifah, 2008).

## 5 Conclusions

The request to suspend the appealed administrative decision under the pretext of judicial revocation in the Saudi Board of Grievances is defined as an urgent request submitted to the Board of Grievances to protect the interests of individuals and employees to whom the decisions of the public administration address, and the suspension of implementation is an exception to the general rule where the administrative decision is issued for its implementation by the employees, except In some cases, this implementation of administrative decisions may have harmful effects. Employees and individuals have the right to request the administrative judge to temporarily suspend the implementation of administrative decisions.

The department concerned with studying the request to stop the implementation of administrative decisions does not respond to it unless there are basic conditions that the law stipulates that they must be met, which is the condition of urgency, and that there are serious reasons for requesting a stay of execution. In the presence of these two conditions, the court has the right to suspend the implementation of the administrative decision through the issuance of a judicial ruling.

In its judicial rulings, the Saudi Board of Grievances emphasizes the temporary nature of the judicial rulings issued in the request to stop the implementation of the decisions of the public administration, like other urgent rulings. It is also a definitive ruling that has the elements and characteristics of a judicial ruling, and therefore it acquires the strength of the thing. That the provision has been adapted in this respect, and thus the time-limited provision affords judicial protection governed by time until a judgment is rendered in the event of annulment, and thus acquires partial authority due to the nature of urgency.

If a judgment is issued rejecting the rescission lawsuit, the judgment issued to stay the execution will not have any effect and become as it was not, but if the judgment issued cancels the administrative decision, this means that the judgment suspending the execution will continue to be in force.

## 6 Recommendations

1. The existence of an explicit legal text stipulating the suspension of the implementation of the contested administrative decision in the case of judicial revocation, without any authority granted to the Board of Grievances in assessing the necessary circumstances to stop the implementation of the decision, as the appeal will have an automatic effect on suspending the implementation of the decision as a general principle, and if the public administration sees the existence of Serious reasons of public interest calling for execution, she has to ask the judge for an order.
2. Reconsidering the name and replacing the "request to suspend the administrative decision" with "request to temporarily suspend the administrative decision" or "request to suspend the decision" so that it does not mix with the issue that is the subject of the case (cancellation case).
3. Addressing the legal regulations related to the request to stay the implementation of decisions related to its procedures, appeal, and the degree of its authority, and not subjecting them to general rules, because failure to address these issues may sometimes lead to slowness in consideration. In cases, this would harm people's interests. Accordingly, the request for a stay of execution is a feature of urgency, so it must be addressed by increasing the number of specialized administrative departments in the Board of Grievances.

## Declaration

### Author contribution statement

Noor Issa Alhendi: Conceived and designed the experiments; Performed the experiments; Analyzed and interpreted the data; Contributed reagents, materials, analysis tools or data; Wrote the paper.

### Funding statement

This research did not receive any specific grant from funding agencies in the public, commercial, or not-for-profit sectors.

### Data availability statement

Data will be made available on request.

### Declaration of interests statement

The authors declare no conflict of interest.

### Additional information

No additional information is available for this paper.

## Footnotes

1 2^nd^ administrative department, timed verdict no. 12//1/2 year 1418h, case no. 611/1/decision year 1418h date 25/7/1418h. 25/11/1997.

2 2nd administrative department, timed verdict no. 19/decision/1/2 year 1420h, case no. 611/1/decision year 1418h, date 15/10/1420h. 2000/1/22.

3 Administrative verdicts and principles set, year 1428h, vol. 4, judicial order no. 148/decision/a/1, year 1428h, 27/12/1428h, case no. (6019/1/decision) year 1427h, 6/1/2008. page 2024.

4 Verdict by the 7^th^ sub-department no. 1/7 year 1417h, 12/11/1996 case no. 1864/1/decision, year 1416h.

5 Verdict by Board of Grievances no. 17/decision/1/4 year 1412h. 15/7/1991.unpublished, Buzaid Aldeen Aljailani, the administrative law, previous ref. page 194.

6 Verdict by the board of Grievances no. 1/decision/8 year 1418h. 14/5/1997.

7 verdict by the Board of Grivances no. 3/1/1410h, 1989/9/30 Mohammad Thnaibat, Hamdi Ajami, the administrative court, previous ref. page 296.

8 Verdict by the Board of Grievances no. 1/decision/1-year 1410h, 1989.case no. 418/1/decision year 1407, unpublished.

9 Decision no. 31/86 year 1400h, case no. 477/a/decision year 1400h, 1980. canonical a regulatory principle, page 64.

10 Court ruling No. 31/2/n for the year 1404 AH in Case No. 913 for the year 1402.1982.
